# Successful treatment of pulmonary invasive fungal infection by *Penicillium* non*‐marneffei* in lymphoblastic lymphoma: case report and literature review

**DOI:** 10.1002/ccr3.1527

**Published:** 2018-05-02

**Authors:** Isabel Ramírez, Alicia Hidrón, Ricardo Cardona

**Affiliations:** ^1^ Division of Infectious Diseases Department of Internal Medicine Hospital Pablo Tobón Uribe Medellín Colombia; ^2^ Division of Infectious Diseases Department of Internal Medicine Universidad de Antioquia Medellín Colombia; ^3^ Division of Infectious Diseases Department of Internal Medicine Universidad Pontificia Bolivariana Medellín Colombia; ^4^ Department of Pathology Hospital Pablo Tobón Uribe Medellín Colombia

**Keywords:** Chemotherapy, immunosuppression, leukemia, lymphoma, *Penicillium* non‐*marneffei*, transplant

## Abstract

*Penicillium* non‐*marneffei* species rarely cause disease in humans and are encountered most commonly in the clinical laboratory as culture contaminants; however, recently they have emerged as opportunistic pathogens in immunocompromised hosts; therefore, it should not be routinely disregarded without a thorough investigation, especially if normally sterile sites are involved.

## Introduction

Members of the genus *Penicillium* are ubiquitous in the environment, encountered in air, soil, and decaying material, and also they have been reported as the second most dominant fungal contaminant in the International Space Station. *Penicillium* spp. have also been described as components of human oral and gut microbiota 1. The conidia become easily aerosolized; this explains their role as environmental contaminants 2. Historically, non‐*marneffei* species rarely cause disease in humans and are encountered most commonly in the clinical laboratory as culture contaminants; however, recently they have emerged as opportunistic pathogens in immunocompromised hosts. Proof of clinical infection requires histological demonstration of tissue invasion.

Herein, we report a case in which a *Penicillium* non‐*marneffei* species pulmonary invasive fungal infection (IFI) was diagnosed synchronously to lymphoblastic lymphoma.

## Case Report

**Figure 1:**
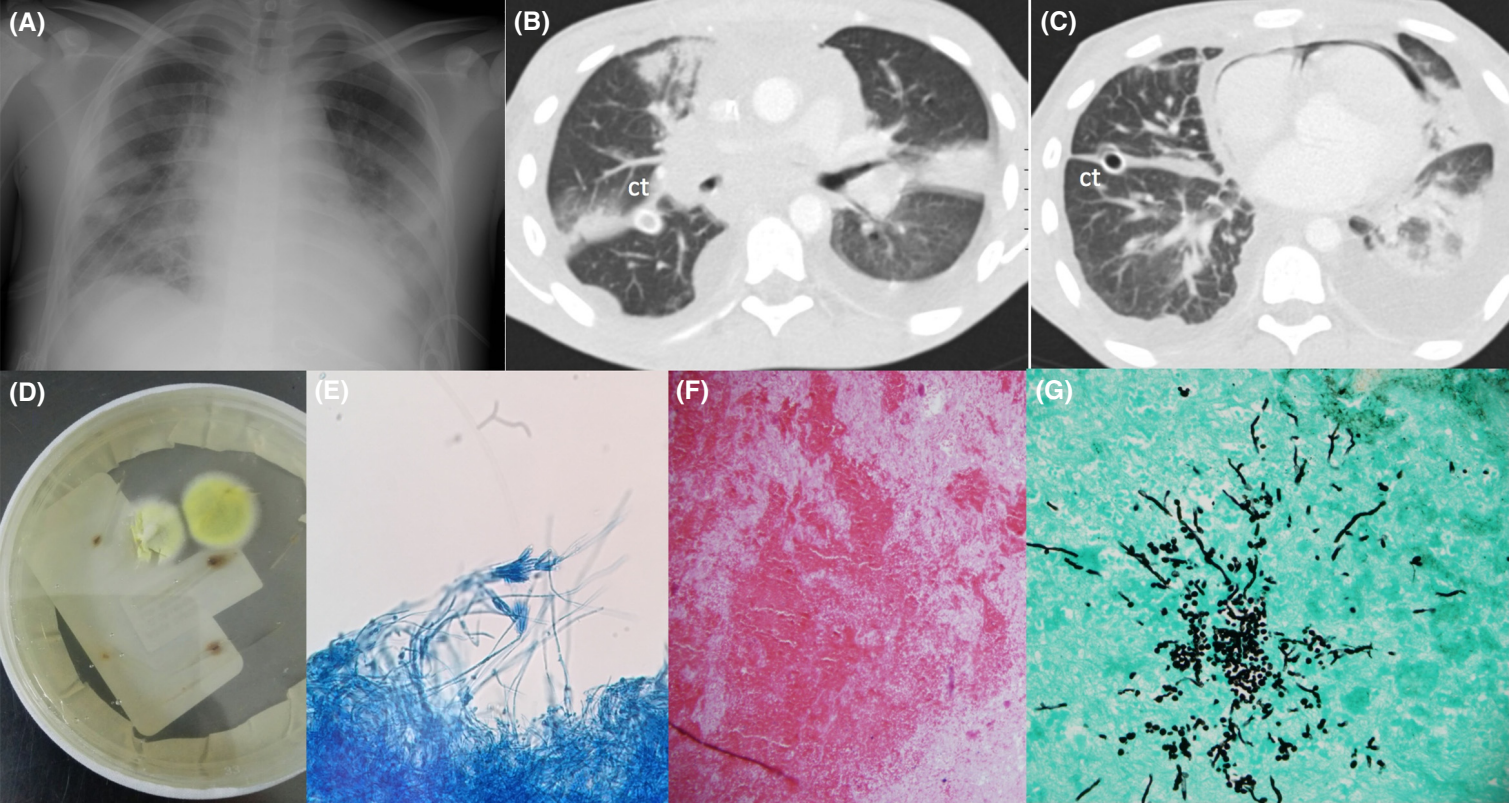
(A) Chest X‐ray showing large mediastinal mass, histologically shown to be malignant lymphoma of lymphoblastic type. (B) Computed tomography showing mediastinal mass surrounding vascular structures and the airway, pulmonary nodule in the right upper lobe, ct: chest tube; (C) pneumomediastinum and pneumopericardium secondary to previous surgery, intrapulmonary infiltrates in the left lower lobe and consolidation in the left upper lobe, and bilateral effusion due to invasive mycosis. (D) Yellow‐green smooth colony growing on the Sabouraud dextrose agar plate at 30°, at day 5. (E) Lactophenol cotton blue stain (x 40). (F) PAS showing necrotic lung and pleural tissue with hemorrhagic areas, accompanied by fibrin (x40). (G) Septate branching fungal mycelia in Grocott methenamine silver (x100).

A 16‐year‐old male was admitted with 4 weeks of systemic symptoms, bilateral pleural and pericardial effusions. A chest X‐ray and CT scan revealed pulmonary nodules and a mediastinal mass (Fig. 1A–C). The patient underwent bilateral thoracoscopy. A lung mass biopsy was performed, and lymphoblastic lymphoma was diagnosed with pathology and flow cytometry. Direct smears of the pulmonary nodules were negative, but on fungal culture (lung tissue was inoculated on Sabouraud dextrose agar at 30°C), a colony of rapidly growing mold was isolated, identified as *Penicillium* spp. (Fig. 1D and E). Multiple attempts to identify species were unsuccessful, including molecular techniques, analysis of sequences of recombinant DNA internal transcribed spacer (ITS 1 and ITS2), and MALDI‐TOF MS. The infection was confirmed with histological sections that stained with hematoxylin and eosin showing necrotic lung and pleural tissue with hemorrhagic areas, and Grocott methenamine silver showed numerous branching hyphae surrounded by suppurative granulomatous inflammation and vascular invasion (Fig. 1F and G). Antifungal susceptibility testing was determined according to CLSI criteria. In vitro susceptibility at 48 h reported minimal inhibitory concentrations for the following antifungals as follows: amphotericin B 1.0 *μ*g/mL and voriconazole 2 *μ*g/mL. The patient completed an 8‐week course of amphotericin B deoxycholate, followed by amphotericin B‐suppressive treatment during each of the remaining seven cycles of chemotherapy, without recurrence of infection. Nine months later, the patient underwent haploidentic hematopoietic stem cell transplantation. Suppressive treatment with amphotericin B was prescribed again, without recurrence of the infection. Up until the present date, 12 months after HSCT, the patient is in remission, without immunosuppression, and without relapse of the infection. We postulate this case represents infection by *Penicillium non‐marneffei* species as the sample was obtained from a surgical specimen in a sterile site, there was no other isolation, and the galactomannan was negative.

## Discussion

*Talaromyces marneffei* causes a typical clinical syndrome in endemic areas (Southeast and eastern regions of Asia) in patients with advanced HIV 3, specific immunodeficiency (autoantibody against interferon‐Gamma) 4, solid organ transplantation 5, 6, 7, 8, and, less commonly, in otherwise healthy individuals 9. This species was recently transferred to the genus *Talaromyces* together with other *Penicillium* species belonging to the subgenus *Biverticillium as T. piceus and Talaromyces purpurogenus* (previously *P. piceum* and *P. purpurogenum*) 10.

*Penicillium*‐like fungi are commonly recovered from clinical samples, in routine hospital air surveys and in clinical practice, and are often encountered as airborne contaminants of culture specimens 11, 12. Usually, *Penicillium* species isolated from samples of non‐AIDS patients are mostly discarded as environmental contaminants. However, in immunosuppressed patients, *non‐marneffei* species are being increasingly recognized as emerging opportunistic pathogens causing invasive fungal infections worldwide, with most reports involving *P*. *citrinum*,* P. digitatum*, and *P. chrysogenum* (Table 1) 13, 14, 15, 16, 17, 18, 19, 20, 21, 22, 23.

**Table 1:** Cases of Penicillium non‐marneffei species infections in hematological malignancy and transplant patients

Ref	Underlying disease	Clinical presentation	Organism and identification	Type of Specimen positive	In Vitro Susceptibility (MIC)	Treatment	Outcome
13	AML 69/F	Pulmonary IFI Pericarditis	*P. citrinum*	Sputum culture, lung tissue pathology postmortem	AMB, ITZ, FCZ, and 5‐FC >32 *μ*g/mL	AMB and ITZ	Died
14	ALL 19/F	Pulmonary IFI	*P. citrinum*	Lung tissue Histopathology postmortem	AMB 12.5 *μ*g/mL MCZ 0.78 *μ*g/mL	FCZ, MCZ 5‐FC	Died
15	AML 44/M	Pulmonary IFI	*T. purpurogenus*	Culture of bronchial lavage x2	NA	AMB	Cured of infection, death within 2 months from septic shock, no autopsy
16	AML 16/M	Pulmonary IFI	*Penicillium* sp.	Tissue cavitation	NA	AMB	Cured
17	AML 44/M	Pulmonary IFI	*P. notatum*	Culture forms bronchoalveolar lavage and lung tissue with fungal angioinvasion	NA	VCZ	Cured
18	AML 12/F	Pulmonary IFI and Hepatic abscess	*P. oxalicum*	Fine‐needle aspiration (FNA) hepatic lesion	AMB <0.03 *μ*g/mL VCZ 2 *μ*g/mL ITZ 0.5 *μ*g/mL ISA 8 *μ*g/mL PCZ 0.125 *μ*g/mL CSP 1 *μ*g/mL	6 weeks PCZ	Survived
19	ALL 40/NA	Disseminated disease	*P. commune*	Postmortem histology	NA	NA	Died
20	ALL/BMT 21/F	Pulmonary IFI Necrotic lung fungus ball	*P. brevicompactum*	Lung tissue and postmortem histopathology	AMB 1.0 *μ*g/mL ITZ 0.5 *μ*g/mL 5‐FC 16 *μ*g/mL	AMB 5‐FC	Died
21	MM/BMT and Plasmocytoma 66/F	Pulmonary IFI	*T. purpurogenus*	Sputum	Not performed	VCZ	Died
Present	Lymphoblastic lymphoma 16/M	Pulmonary IFI	*Penicillium* sp.	Lung tissue pulmonary biopsy	AMB 1.0 *μ*g/mL, ITZ 0.25, VCZ 1 *μ*g/mL	AMB	Cured
22	Lung transplant 56/M	Pulmonary IFI	*P. chrysogenum*	BAL	AMB 16 *μ*g/mL VCZ 0.25 *μ*g/mL CSP 0.19 *μ*g/mL PCZ 0.25 *μ*g/mL	PCZ and CSP	Died
23	Kidney transplantation 37/M	Fungemia	*P. chrysogenum*	Blood culture	AMB <0.5 mg/L ITZ <0.5 mg/L 5‐FC <0.5 mg/L	Postmortem diagnosis	Died

ALL, acute lymphoblastic leukemia; BMT, bone marrow transplant; AML, acute Myeloid leukemia; MM, multiple myeloma; IFI, invasive fungal infection; NA, not available; BAL, bronchoalveolar lavage; AMB, amphotericin B deoxycholate; FCZ, fluconazole; VCZ, voriconazole; ITZ, itraconazole; PCZ, posaconazole; CSP, caspofungin; 5‐FC, 5‐flucytosine; MCZ, miconazole.

The clinical presentation of Penicilliosis caused by non‐*marneffei* species reported to date is different to that reported for *T. marneffei* species; although pulmonary involvement is common in both, non*‐marneffei* species, particularly in patients with hematological malignancies, usually cause disseminated infection in the setting of invasive pulmonary fungal infection, with high fatality rates.

In patients with hematological malignancies, they have emerged as potential opportunistic agents, mainly described in acute lymphoblastic and myeloid leukemia.

Pulmonary invasive fungal infection involving *P. citrinum*,* P. purpurogenus, P. notatum, P. brevicompactum, P. oxalicum, and P. commune* has been reported 13, 14, 15, 16, 17 *;* including pericardial involvement 13, coinfection with *P. jirovecii* 17 *,* pulmonary and hepatosplenic involvement 18, and disseminated disease 19. In patients undergoing transplantation with hematopoietic progenitors, *P. brevicompactum* has been described causing invasive pulmonary infection 20, and, after autologous bone marrow transplantation for multiple myeloma, pulmonary infection due to *T. purpurogenus* has been described 21. In many cases, the diagnosis was made postmortem with an attributed mortality of 62% 13, 14, 19. In summary, according to the review, the most prevalent species causing infection in immunosuppressed patients is *P. chrysogenum*, which causes systemic and disseminated disease with invasive pulmonary infection. The susceptibility profile is not predictable and may vary according to isolates; some have high MICs to azoles and amphotericin B.

Confirming a diagnosis of Penicilliosis can be difficult by conventional phenotypic methods. Identification at the species level remains challenging, and the high number of species in these genera makes this task even more difficult 1. The use of molecular methods, however, provides a rapid and relatively simple method for the identification of *Penicillium* species. Matrix‐assisted laser desorption ionization time‐of‐flight mass spectrometry (MALDI‐TOF MS) has limitations for identification of dimorphic fungi but may identify species of *Penicillium* and differentiate *T. marneffei* from non‐*marneffei* species as *P. brevicompactum* and *P. chrysogenum*; however, the scores were below the cutoff required for species identification. This can be explained by the limited number of spectra for the two species so that expansion of MALDI‐TOF MS databases is needed 24.

Standard treatment for non‐*marneffei* species has not yet been established; antifungal susceptibility data for clinically available antifungal agents and treatment options for infections caused by *Penicillium* species are also poorly understood, aside from data published for *T. marneffei*. In a recent study of 118 clinical isolates, (mainly from the respiratory tract/human bronchoalveolar lavage) terbinafine (TRB) and the echinocandins showed the best in vitro activity against *Penicillium* species with MIC <0.03 *μ*g/mL for TRB, 0.06 *μ*g/mL for caspofungin and anidulafungin, and 0.125 for micafungin; amphotericin B showed intermediate activity with MIC of 2 *μ*g/mL, and azoles revealed variable activity with MIC ranges of 0.5 *μ*g/mL for posaconazole and 2 *μ*g/mL for voriconazole and itraconazole 25.

In conclusion, *Penicillium* spp. isolates in immunosuppressed patients should not be routinely disregarded without a thorough investigation, especially if normally sterile sites are involved.

## Conflict of Interest

The authors have no conflict of interest to declare.

## Authorship

IR: contributed to the design, drafted, and revised the manuscript. AH: contributed to the design, drafted, and revised the manuscript. RC: contributed to the design, drafted, and revised the manuscript. All the authors: approved the submitted and final versions.

## References

[1] Ghannoum, M. A. , R. J. Jurevic , P. K. Mukherjee , F. Cui , M. Sikaroodi , A. Naqvi , et al. 2010 Characterization of the oral fungal microbiome (mycobiome) in healthy individuals. PLoS Pathog. 6:e1000713. 20072605 10.1371/journal.ppat.1000713 PMC2795202

[2] Sautour, M. , I. Fournel , F. Dalle , C. Calinon , C. L'Ollivier , M. Goyer , et al. 2012 Dynamics of fungal colonization in a new medical mycology laboratory. J. Mycol. Med. 22: 14–20. 23177809 10.1016/j.mycmed.2011.11.001

[3] Anitnori, S. , E. Gianelli , C. Bonaccorso , L. Ridolfo , F. Croce , S. Sollima , et al. 2006 Disseminated *Penicillium marneffei* infection in an HIV‐positive Italian patient and a review of cases reported outside endemic regions. J. Travel. Med. 13:181–188. 16706952 10.1111/j.1708-8305.2006.00039.x

[4] Tang, B. S. , J. F. Chan , M. Chen , O. T. Tsang , M. Y. Mok , R. W. Lai , et al. 2010 Disseminated penicilliosis, recurrent bacteremic nontyphoidal salmonellosis, and burkholderiosis associated with acquired immunodeficiency due to autoantibody against gamma interferon. Clin. Vaccine Immunol. 17:1132–1138. 20445006 10.1128/CVI.00053-10 PMC2897261

[5] Chan, Y. H. , K. M. Wong , K. C. Lee , P. H. Kwok , W. L. Chak , K. S. Choi , et al. 2004 Pneumonia and mesenteric lymphadenopathy caused by disseminated *Penicillium marneffei* infection in a cadaveric renal transplant recipient. Transpl. Infect Dis. 6:28–32. 15225224 10.1111/j.1399-3062.2004.00038.x

[6] Stathakis, A. , K. P. Lim , P. Boan , M. Lavender , J. Wrobel , M. Musk , et al. 2015 *Penicillium marneffei* infection in a lung transplant recipient. Transpl. Infect Dis. 17:429–434. 25809145 10.1111/tid.12377

[7] Hart, J. , J. R. Dyer , B. M. Clark , D. G. McLellan , S. Perera , P. Ferrari , et al. 2012 Travel‐related disseminated *Penicillium marneffei* infection in a renal transplant patient. Transpl. Infect Dis. 14:434–439. 22188555 10.1111/j.1399-3062.2011.00700.x

[8] Chan, J. F. , S. K. Lau , K. Y. Yuen , and P. C. Woo . 2016 *Talaromyces* (*Penicillium*) *marneffei* infection in non‐HIV‐infected patients. Emerg. Microbes. Infect. 5:e19. 26956447 10.1038/emi.2016.18 PMC4820671

[9] Saadiah, S. , A. H. Jeffrey , and A. L. Mohamed . 1999 *Penicillium marneffei* infection in a non aids patient: first case report from Malaysia. Med. J. Malaysia 54:264–266. 10972040

[10] Samson, R. A. , N. Yilmaz , J. Houbraken , H. Spierenburg , K. A. Seifert , S. W. Peterson , et al. 2011 Phylogeny and nomenclature of the genus *Talaromyces* and taxa accommodated in *Penicillium* subgenus *biverticillium* . Stud. Mycol. 70:159–183. 22308048 10.3114/sim.2011.70.04 PMC3233910

[11] da Silva, A. , J. C. Porto , J. L. Da Silva , K. F. Morais , F. A. Coelho , Lopes T. de Sousa , et al. 2016 Evaluation of disinfectants for elimination of fungal contamination of patient beds in a reference hospital in Piauí, Brazil. Environ. Monit. Assess. 188:644. 27796829 10.1007/s10661-016-5654-z

[12] Okten, S. , and A. Asan . 2012 Airborne fungi and bacteria in indoor and outdoor environment of the Pediatric Unit of Edirne Government Hospital. Environ. Monit. Assess. 184:1739–1751. 21611848 10.1007/s10661-011-2075-x

[13] Mok, T. , A. P. Koehler , M. Y. Yu , D. H. Ellis , P. J. Johnson , and N. W. Wickham . 1997 Fatal *Penicillium citrinum* pneumonia with pericarditis in a patient with acute leukemia. J. Clin. Microbiol. 35:2654–2656. 9316926 10.1128/jcm.35.10.2654-2656.1997 PMC230029

[14] Mori, T. , M. Matsumura , T. Kohara , Y. Watanabe , T. Ishiyama , Y. Wakabayashi , et al. 1987 A fatal case of pulmonary penicilliosis. Jpn. J. Med. Mycol. 28:341–348.

[15] Breton, P. , P. Germaud , O. Morin , A. F. Audouin , N. Milpied , and J. L. Harousseau . 1998 Rare pulmonary mycoses in patients with hematologic diseases. Rev. Pneumol. Clin. 54:253–257. 9894280

[16] Shamberger, R. C. , H. J. Weinstein , H. E. Grier , and R. H. Levey . 1985 The surgical management of fungal pulmonary infections in children with acute myelogenous leukemia. J. Pediatr. Surg. 20:840–844. 3866846 10.1016/s0022-3468(85)80052-x

[17] Shokouhi, S. , S. Tehrani , and M. Hemmatian . 2016 Mixed pulmonary infection with *Penicillium notatum* and *Pneumocystis jiroveci* in a patient with acute myeloid leukemia. Tanaffos 15:53–56. 27403180 PMC4937763

[18] Chowdhary, A. , S. Kathuria , K. Agarwal , N. Sachdeva , P. K. Singh , S. Jain , et al. 2014 Voriconazole‐resistant *Penicillium oxalicum*: an emerging pathogen in immunocompromised hosts. Open Forum Infect. Dis. 1:ofu029. 25734109 10.1093/ofid/ofu029 PMC4281804

[19] Huang, S. N. 1963 Acute disseminated penicilliosis. Report of a case and review of the pertinent literature. Am. J. Clin. Pathol. 39:167–174. 13955352 10.1093/ajcp/39.2.167

[20] de la Cámara, R. , I. Pinilla , E. Muñoz , B. Buendía , J. L. Steegmann , and J. M. Fernández‐Rañada . 1996 *Penicillium brevicompactum* as the cause of a necrotic lung ball in an allogeneic bone marrow transplant recipient. Bone Marrow Transplant. 18:1189–1193. 8971395

[21] Atalay, A. , A. N. Koc , G. Akyol , N. Cakir , L. Kaynar , and A. Ulu‐Kilic . 2016 Pulmonary infection caused by *Talaromyces purpurogenus* in a patient with multiple myeloma. Infez. Med. 24:153–157. 27367328

[22] Geltner, C. , C. Lass‐Flör , H. Bonatti , L. Müller , and I. Stelzmüller . 2013 Invasive pulmonary mycosis due to *Penicillium chrysogenum*: a new invasive pathogen. Transplantation 95:e21–e23. 23423272 10.1097/TP.0b013e31827ff214

[23] Swoboda‐Kopec, E. , M. M. Wroblewska , A. Rokosz , and M. Luczak . 2003 Mixed bloodstream infection with *Staphylococcus aureus* and *Penicillium chrysogenum* in an immunocompromised patient: case report and review of the literature. Clin. Microbiol. Infect. 9:1116–1117. 14616728 10.1046/j.1469-0691.2003.00718.x

[24] Lau, S. K. , C. S. Lam , A. H. Ngan , W. N. Chow , A. K. Wu , D. N. Tsang , et al. 2016 Matrix‐assisted laser desorption ionization time‐of‐flight mass spectrometry for rapid identification of mold and yeast cultures of *Penicillium marneffei* . BMC Microbiol. 16:36. 26965891 10.1186/s12866-016-0656-0 PMC4787007

[25] Guevara‐Suarez, M. , D. A. Sutton , J. F. Cano‐Lira , D. García , A. Martin‐Vicente , N. Wiederhold , et al. 2016 *Penicillium*‐like fungi from clinical samples in the USA and their antifungal susceptibility. J. Clin. Microbiol. 54:2155–2161. 27280422 10.1128/JCM.00960-16 PMC4963513

